# The association between body mass index and health and economic outcomes in Brazil

**DOI:** 10.1186/s13098-018-0322-9

**Published:** 2018-03-16

**Authors:** Ian Kudel, Jefferson S. Alves, Thiago de Menezes Goncalves, Kristjan Kull, Emil Nørtoft

**Affiliations:** 1 Kantar Health, New York, USA; 2 Novo Nordisk Brazil, São Paulo, Brazil; 3 Novo Nordisk, São Paulo, Region Latin America Brazil; 4 Global Market Access, Novo Nordisk A/S, vandtårnsvej 112, 2860 Søborg, Denmark

**Keywords:** Obesity, Body mass index, Quality of life, Direct costs, Indirect costs

## Abstract

**Background:**

Obesity is associated with significant physical, psychosocial and economic burden globally. In Brazil, almost 50% of the population is either overweight or obese. The prevalence of morbid obesity increased by 255% between 1975 and 2003. The current study sought to quantify the relationship between weight status and health outcomes.

**Methods:**

Data from three waves (2011, 2012, and 2015) of the Brazil National Health and Wellness Survey, an Internet-based survey administered to a demographically diverse sample of Brazilian adults, were used. Body mass index category was calculated based on self-reported height and weight and respondents were categorized into five groups (normal, overweight, obese class I, obese class II, obese class III; n = 34,254). Multivariable analyses, controlling for sociodemographic variables and health history, tested the association with body mass index group and outcomes including health status (Medical Outcomes Study Short Form 12-Item Health Survey version 2/Medical Outcomes Study Short Form 36-Item Health Survey version 2), work productivity (Work Productivity and Activity Impairment-General Health Questionnaire), and costs associated with work impairment (indirect costs), self-reported healthcare resource use and associated direct costs.

**Results:**

Overall, 53.6% of the surveyed Brazilian population reported being overweight or obese. In virtually all the analyses, increasing body mass index group was associated with significant and progressively worse outcomes. Most notable was the finding that hospitalization costs were over twice as high (R$3141.84 vs. R$1349.60) and indirect costs were nearly double (R$1656.80 vs. R$884.15) for obesity class III than for normal body mass index respondents.

**Conclusions:**

Obesity rates in Brazil are considerable and, from a patient and societal perspective, increasingly burdensome, thereby highlighting the need for stakeholders to prioritize strategies for weight management interventions.

## Background

Obesity is associated with a physical, psychosocial and economic burden globally. According to the World Health Organization, obesity is defined by having a body mass index (BMI) ≥ 30 kg/m^2^, with the degree of obesity defined as class I (BMI 30–34.9 kg/m^2^), class II (BMI 35.0–39.9 kg/m^2^), and class III (BMI ≥ 40 kg/m^2^) [1]. Over the last few decades, the worldwide obesity epidemic has continued to grow at a high rate, with the age-standardized prevalence nearly doubling between 1980 and 2008 [2].

In Brazil, the prevalence of morbid obesity increased by 255% between 1975 and 2003, but the prevalence of obesity was lower at a 152% increase [3]. The greatest increase of morbid obesity was observed in the Southeast, followed by the South [3] and among various subpopulations, including adolescents [4], indigenous people [5], and women [6]. Additionally, over 13% of Brazilian adults are obese [6]. Obesity has also been shown to be a risk factor for a variety of diseases such as cardiovascular disease, cancer, type 2 diabetes (T2D), osteoarthritis, non-alcoholic fatty liver disease, sleep apnea, and psychiatric conditions [7–9]. Indeed, it is estimated that 20% of all cancer cases, independent of diet, can be attributed to obesity [10].

Additionally, obesity and the comorbidities associated with the condition (e.g. hypertension, diabetes) have been linked to a shorter life expectancy [11, 12], with men and women 20–30 years of age estimated to lose 13–20 and 5–8 years of life, respectively [11]. Further, for non-smokers over the age of 40 years men and women are expected to lose 3.1 and 3.3 years of life, respectively [12].

The burden associated with obesity also extends to psychosocial, symptom, and work-related domains. Evidence suggests an association between increasing BMI and greater pain [13], fatigue and sleep disorders [14, 15], as well as depressed mood [16] and broader impairments in health-related quality of life (HRQoL) [17, 18]. Global studies have also reported a consistent association between obesity and impairments in work productivity measures, including absenteeism and presenteeism [19–22].

Obesity has also been found to have substantial societal costs globally. For example, a European-based review found that obesity-related healthcare costs exceed €10 billion and that obesity is a substantial burden in the majority of the European countries, representing 0.09–0.61% of gross domestic product [23]. Similar findings have been reported among other developed countries, most notably the United States (US), where one study reported obesity to increase annual medical costs by $2741 [24]. Similar findings of high economic burden exist within Latin America. For example, a recent Mexican study found that obesity-related disease was estimated to be responsible for $US 806 million in costs in 2010, and is predicted to rise to $US 1.2 billion by 2030 [25].

Not surprisingly, many of the same trends are seen in Brazil [26]. Torres and colleagues reported a significant association between obese weight status and impaired HRQoL among individuals in Niteroi [27], while Turco reported impaired HRQoL and sleep quality among adolescences with obesity in Sao Paulo [28]. The societal costs of obesity are also great. Using data from 2001 it was found that more than 1 million workdays, an indirect cost, was lost because of obesity-related factors [29]. The direct costs to the Brazilian society are also high. Bahia et al. [30] found, using data collected from 2008 to 2010, that the estimated cost of all diseases related to overweight and obesity was $2.1 billion in 1 year; $1.4 billion was related to hospitalizations and $679 million was related to ambulatory procedures. These costs are expected to increase from $5.8 billion in 2010 to $10.1 billion by 2050 [31]. Furthermore, studies have established higher medication costs among patients with obesity [32], while a recent study of 2201 employees of a Brazilian airline found that obesity was associated with higher direct healthcare costs, with each BMI point increasing annual costs by $17 US dollars [33].

Overall however, large-scale survey data on the relationship between obesity and health outcomes in Brazil is limited. Whereas previous studies have documented the changing epidemiology and the health consequences of obesity [31], few have examined the patient-reported effects of obesity, such as health-related quality of life and impairment in daily activities. The objective of the current study is to quantify the relationship between BMI and health outcomes, including health status, work productivity, indirect costs, healthcare resource use, and direct costs.

## Methods

### Data source

Data for this research study come from 3 years (2011, 2012, and 2015; n = 36,000) of the Brazil National Health and Wellness Survey (NHWS), an Internet-based survey administered to a nationwide sample of Brazilian adults, aged 18 years and older. Survey results are collected using a dual methodology of Internet and, for elderly respondents, computer assisted web interviewing. A random stratified sample, based on gender, age, and socioeconomic status, was used to ensure the demographic composition of the sample is representative of the Brazilian adult population. For this purpose, gender and age stratification are based on statistics from the International Database of the US Census Bureau, and socioeconomic status is based on data from Brazil’s Institute of Geography and Statistics. The NHWS received approval from the Essex Institutional Review Board (IRB). All respondents provided informed consent prior to participating, and they were only known by a unique identifier.

### Sample

All respondents with non-missing weight data were included (n = 35,501). Underweight respondents (i.e., body mass index < 18.5; n = 1206) were excluded from the analyses.

### Measures

#### Weight status: BMI

BMI was calculated based on participant responses on items asking “What is your height?” and “What is your weight?” BMI was the key independent variable and participants were divided into five groups: normal weight range (BMI 18.5–25), overweight (BMI 25–29.99), obese class I (BMI 30–34.99), obese class II (BMI 35–39.99) and obese class III (BMI 40+).

#### Demographics and health characteristics

Demographic and health characteristics were used as covariates in multivariable analyses. They included age, sex (male or female), marital status (married/living with partner or not-married), education (university degree vs. less than university degree), household income, smoking status (currently smokes, former smoker vs. never smoker), alcohol use (currently drink vs. do not currently drink), exercise behavior (number of days exercised in the past month).

#### Charlson comorbidity index

The Charlson comorbidity index (CCI) was used to control for a range of health conditions in the multivariable analyses. It incorporates a range of conditions including HIV/AIDS, metastatic tumor, lymphoma, leukemia, any tumor, moderate/severe renal disease, hemiplegia, diabetes, mild liver disease, ulcer disease, connective tissue disease, chronic pulmonary disease, dementia, cerebrovascular disease, peripheral vascular disease, myocardial infarction, congestive heart failure, and diabetes with end organ damage. A higher score is indicative of greater comorbidity burden [34].

#### Region

Participants reported their region of residence. Options include North, Northwest, Center-West, Southeast and South.

#### Health status

Health status was assessed using the Medical Outcomes Study Short Form 36-Item Health Survey version 2 (SF-36v2) [35] or the Medical Outcomes Study Short Form 12-item Health Survey version 2 (SF-12v2). The items for both measures map onto eight health domains: physical functioning, physical role limitations, bodily pain, general health, vitality, social functioning, emotional role limitations, and mental health. In addition, there are two component summary scores derived from these questions, which were used in the analyses for the current study: the physical component summary (PCS) and the mental component summary (MCS). The summary scores were used in this study. They are calculated using a norm-based scoring algorithm that yields scores ranging from 0 to 100. Further it allows for scores to be interpreted relative to population (i.e., mean of 50). The minimally important difference (MID), the smallest change in an outcome that a patient would identify as important, for the PCS and MCS is 3 points [36–38].

The information was also used to derive the Short Form 6-Dimensions (SF-6D), a preference-based health utility index [35]. Scores range from 0 (a health state equivalent to death) to 1 (a health state equivalent to perfect health). Past research has suggested the MID for the SF-6D is 0.03 points [36–38].

#### Work productivity

Work productivity was assessed using the Work Productivity and Activity Impairment-General Health (WPAI-GH) Questionnaire, a 6-item validated instrument which consists of four metrics: absenteeism (the percentage of work time missed because of one’s health in the past 7 days), presenteeism (the percentage of impairment experienced while at work in the past 7 days because of one’s health), overall work productivity loss (an overall impairment estimate that is a combination of absenteeism and presenteeism), and activity impairment (the percentage of impairment in daily activities because of one’s health in the past 7 days) [39]. Only respondents who reported being full-time or part-time employed provided data for absenteeism, presenteeism, and overall work impairment. All respondents provided data for activity impairment. Scores for each subscale range from 0 to 100, but unlike the MCS and PCS they are no norm-based scores.

Absenteeism is calculated by dividing the number of work hours a patient missed in the past week because of his or her health by the total number of hours the total number of hours they were expected to work (the number of hours they did work plus the number of hours they missed because of their health), and then converting the proportion into a percentage. For example, if a patient missed 10 h and worked 30 h, then absenteeism would be 25% (10 ÷ (10 + 30) = 0.25).

Presenteeism is based on participants’ rating (from 0 to 10) of impairment experienced while at work over the previous 7 days. That score was then multiplied by 10 to create a percentage. For example, if a participant reported her level of impairment was a “2,” it would be converted to a presenteeism level of 20%.

Overall work impairment was measured by combining absenteeism and presenteeism to determine the total percentage of missed time. Activity impairment was measured by a patient’s response to the level of impairment experienced in daily activities in the past 7 days (from 0 to 10), which was then multiplied by 10 to create a percentage, ranging from 0 to 100%.

#### Healthcare resource use

Healthcare utilization was based on participants recall of the number of healthcare provider visits, the number of emergency room (ER) visits (“*how many times have you been to the ER for your own medical condition in the past 6* *months?*”), and the number of times hospitalized (*“how many times have you been hospitalized for your own medical condition in the past 6* *months?”*) in the past 6 months. The phrasing “own medical condition” was used to ensure that participants’ are recalling their own use of the resource rather than trips to accompany a friend or relative. The phrasing is intentionally vague so that all medical conditions are included.

#### Annual costs

The cost for an average ER visit, hospitalization, and physician visit were obtained from the various sources. Private physician costs were obtained from Classificação Brasileira Hierarquizada de Procedimentos Médicos (CBHPM) and public costs were obtained from Sistema de Gerenciamento da Tabela de Procedimentos, Medicamentos e OPM do SUS (SIGTAP). Private hospitalization costs, which reflects the average cost for a general episode, were obtained from União Nacional das Instituições de Autogestão em Saúde (Unidas) and public costs were obtained from Autorização de Internação Hospitalar from: Ministério da Saúde. Secretaria de Atenção à Saúde (SAS): Sistema de Informações Hospitalares no SUS (SIH/SUS; AIH). For each respondent, the number of each type of visit was multiplied by two to project to the annual number of visits and then multiplied by its average cost. Next, each of those figures was summed to a total direct cost value for each respondent.

Indirect costs for employed respondents were calculated using average annual salaries that were derived from the Organization for Economic Cooperation and Development [40]. The percentage of overall work impairment was multiplied by annual salary to estimate work lost due to health.

### Statistical analyses

#### Descriptive analyses

Participants demographic and health characteristics for the overall sample and each weight class were derived. Count and percentages were derived for categorical variables and mean and standard deviations were calculated for continuous variables. Mean, standard deviation, and ranges for the outcome variables for the entire sample were calculated.

#### Multivariable analyses

A series of generalized linear models (GLMs) were used to determine the association between BMI group and outcome variables controlling for sociodemographic and health-related variables. A normal distribution was specified for health status variables whereas, due to pronounced skewing, a negative binomial distribution and log-link function were specified for productivity, healthcare resource use, and cost variables. The reference group was the normal BMI group and to facilitate interpretation adjusted means (least-squares means presented at the mean of the covariates) are reported. The same covariates were included in each model. They included age, gender, race, marital status, education, income, exercise, smoking, alcohol use, and CCI.

## Results

### Descriptive statistics

Among all respondents (n = 34,254), a total of 46.4% were normal weight, 34.9% were overweight, and the remaining 18.7% were obese (12.9% were obese class I, 3.7% were obese class II, and 2.1% were obese class III). Thus, overall 53.6% of the surveyed Brazilian population was overweight or obese.

Participants’ location, sociodemographic variables and health characteristics for the overall sample and each weight class were derived (Table 1). The majority of the respondents where from the Southeast, male, white, middle-aged, college educated, employed, middle-class [B1–B2], and married. More than half reported exercising more than one time per month, never smoking, consuming less than one alcoholic beverage per month, and, based on the number of comorbid conditions reported, generally healthy. The distribution of demographics, with only slight variations, was similar across all BMI groups.

**Table 1:** Demographics and health history differences across BMI classes among all adults

BMI group	Total	Normal (18.5 to < 25)	Overweight (25 to < 30)	Obese I (30 to < 35)	Obese II (35 to < 40)	Obese III (40+)
		(n = 15,893)	(n = 11,960)	(n = 4423)	(n = 1269)	(n = 707)
	Categorical covariates					
Region: n (%)						
North	768 (2.2%)	365 (2.3%)	260 (2.2%)	100 (2.3%)	30 (2.4%)	13 (1.8%)
Northeast	4885 (14.3%)	2381 (15.0%)	1671 (14.0%)	586 (13.3%)	155 (12.2%)	92 (13.0%)
Center-West	2391 (7.0%)	1122 (7.1%)	858 (7.2%)	292 (6.6%)	80 (6.3%)	39 (5.5%)
Southeast	20,420 (59.6%)	9269 (58.4%)	7181 (60.1%)	2732 (61.8%)	793 (62.5%)	445 (63.0%)
South	5749 (16.8%)	2733 (17.2%)	1979 (16.6%)	709 (16.0%)	211 (16.6%)	117 (16.6%)
Sex: n (%)						
Male	17,540 (51.2%)	7410 (46.6%)	6908 (57.8%)	2372 (53.6%)	545 (42.9%)	305 (43.1%)
Female	16,712 (48.8%)	8483 (53.4%)	5052 (42.2%)	2051 (46.4%)	724 (57.1%)	402 (56.9%)
Race: n (%)						
White	21,916 (64.0%)	10,025 (63.1%)	7688 (64.3%)	2903 (65.6%)	828 (65.2%)	472 (66.8%)
Black	2714 (7.9%)	1215 (7.6%)	979 (8.2%)	347 (7.8%)	110 (8.7%)	63 (8.9%)
Amarelo	867 (2.5%)	487 (3.1%)	259 (2.2%)	87 (2.0%)	23 (1.8%)	11 (1.6%)
Pardo	8215 (24.0%)	3878 (24.4%)	2882 (24.1%)	1023 (23.1%)	283 (22.3%)	149 (21.1%)
Indigena	209 (0.6%)	109 (0.7%)	60 (0.5%)	25 (0.6%)	8 (0.6%)	7 (1.0%)
Decline to answer	331 (1.0%)	179 (1.1%)	92 (0.8%)	38 (0.9%)	17 (1.3%)	5 (0.7%)
Education: n (%)						
High school or less	12,201 (35.6%)	5654 (35.6%)	4235 (35.4%)	1564 (35.4%)	461 (36.3%)	287 (40.6%)
At least some college	22,051 (64.4%)	10,239 (64.4%)	7725 (64.6%)	2859 (64.6%)	808 (63.7%)	420 (59.4%)
Socioeconomic status: n (%)						
A1–A2 [upper class]	4351 (12.7%)	1844 (11.6%)	1634 (13.7%)	612 (13.8%)	172 (13.6%)	89 (12.6%)
B1–B2 [middle class]	19,321 (56.4%)	8715 (54.8%)	6905 (57.7%)	2608 (64.4%)	705 (55.6%)	388 (54.9%)
C1 [lower middle class]	6967 (20.3%)	3382 (21.3%)	2314 (19.3%)	832 (18.8%)	282 (22.2%)	157 (22.2%)
C2 [skilled working class]	2730 (8.0%)	1449 (9.1%)	850 (7.1%)	283 (6.4%)	92 (7.2%)	56 (7.9%)
D [lower working class]	812 (2.4%)	469 (3.0%)	231 (1.9%)	83 (1.9%)	15 (1.2%)	14 (2.0%)
E [lowest income earners]	71 (0.2%)	34 (0.2%)	26 (0.2%)	5 (0.1%)	3 (0.2%)	3 (0.4%)
Marital status: n (%)						
Single	15,236 (44.5%)	8169 (51.4%)	4695 (39.3%)	1574 (35.6%)	500 (39.4%)	298 (42.1%)
Married	19,016 (55.5%)	7724 (48.6%)	7265 (60.7%)	2849 (64.4%)	769 (60.6%)	409 (57.9%)
Exercise 20+ min ≥ 1 times in past month: n (%)						
Exercise 0 times	13,729 (40.1%)	5938 (37.4%)	4675 (39.1%)	2039 (46.1%)	676 (53.3%)	401 (56.7%)
Exercise ≥ 1 times	20,523 (59.9%)	9955 (62.6%)	7285 (60.9%)	2384 (53.9%)	593 (46.7%)	306 (43.3%
Smoking status: n (%)						
Currently smokes	4290 (12.5%)	2024 (12.7%)	1515 (12.7%)	504 (11.4%)	159 (12.5%)	88 (12.4%)
Trying to quit smoking	2002 (5.8%)	901 (5.7%)	745 (6.2%)	255 (5.8%)	61 (4.8%)	40 (5.7%)
Former smoker	7762 (22.7%)	3053 (19.2%)	2838 (23.7%)	1316 (29.8%)	386 (30.4%)	169 (23.9%)
Never smoked	20,198 (59.0%)	9915 (62.4%)	6862 (57.4%)	2348 (53.1%)	663 (52.2%)	410 (58.0%)
Alcohol consumption: n (%)						
Alcohol ≤ 1 time	28,914 (84.4%)	13,606 (85.6%)	9831 (82.2%)	3726 (84.2%)	1118 (88.1%)	633 (89.5%)
Alcohol ≥ 2–3 times	5338 (15.6%)	2287 (14.4%)	2129 (17.8%)	697 (15.8%)	151 (11.9%)	74 (10.5%)
Labor force participation: n (%)						
Not in labor force	7949 (23.2%)	3761 (23.7%)	2730 (22.8%)	1014 (22.9%)	281 (22.1%)	163 (23.1%)
In labor force	26,303 (76.8%)	12,132 (76.3%)	9230 (77.2%)	3409 (77.1%)	988 (77.9%)	544 (76.9%)
	Continuous covariates					
CCI: mean ± SD	0.34 ± 0.91	0.30 ± 0.88	0.33 ± 0.86	0.40 ± 0.96	0.45 ± .96	0.53 ± 1.62
Age: mean ± SD	40.67 ± 15.44	38.22 ± 15.97	43.00 ± 15.15	42.88 ± 13.94	41.98 ± 13.17	39.95 ± 12.11

*BMI* body mass index, *CCI* Charlson comorbidity index, *SD* standard deviation

### Multivariable analyses

#### Health status

**Fig. 1:**
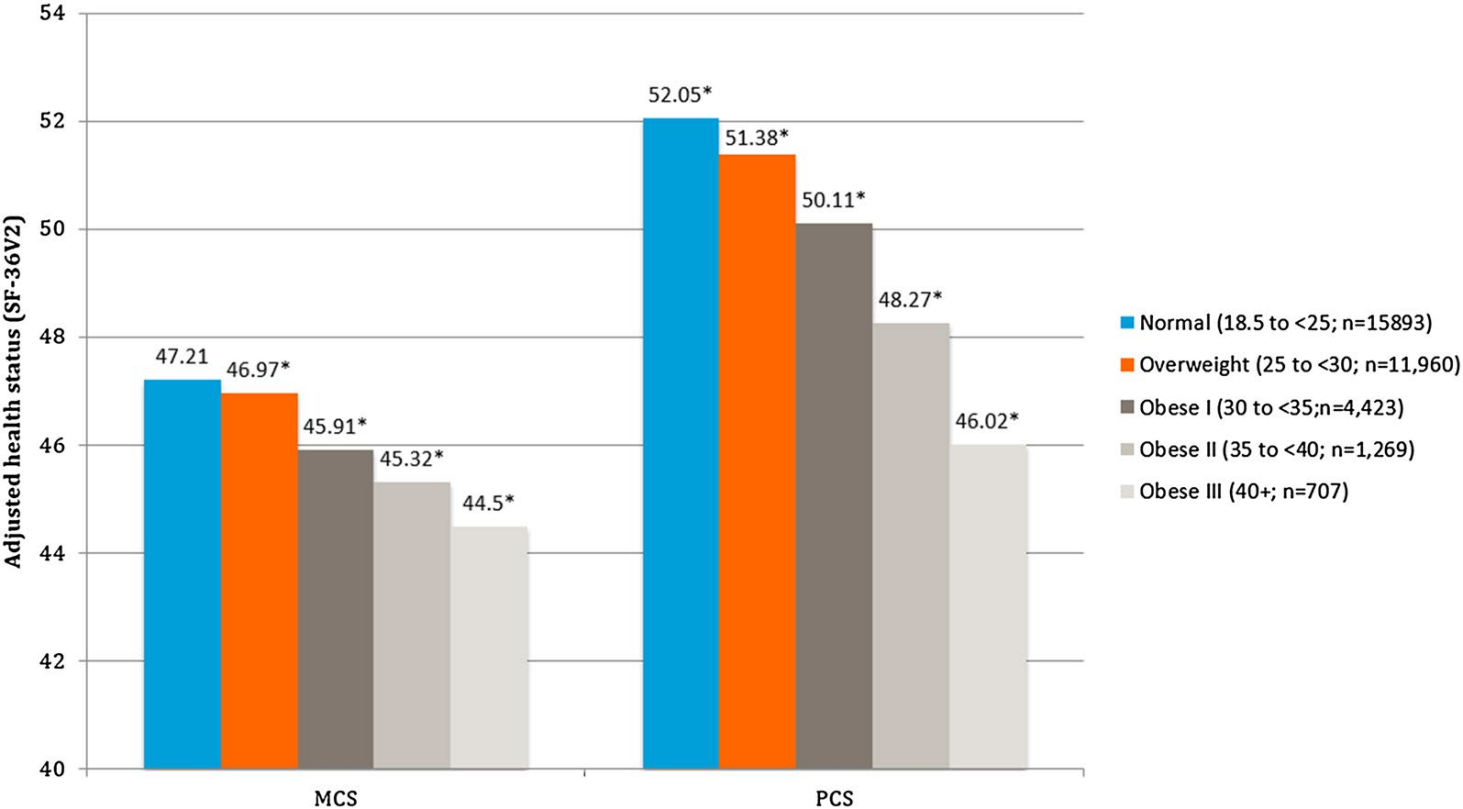
Adjusted mean health status (SF-36v2) scores by BMI class among all respondents. Normal weight: 18.5–24.9 kg/m^2^; overweight: 25–29.9 kg/m^2^; obese class I: 30–34.9 kg/m^2^; obese class II: 35–39.9 kg/m^2^; obese class III: 40 kg/m^2^. *The mean is significantly different than normal-weight group. All models controlled for age, gender, race, marital status, education, income, exercise, smoking, alcohol use, and Charlson comorbidity index

**Fig. 2:**
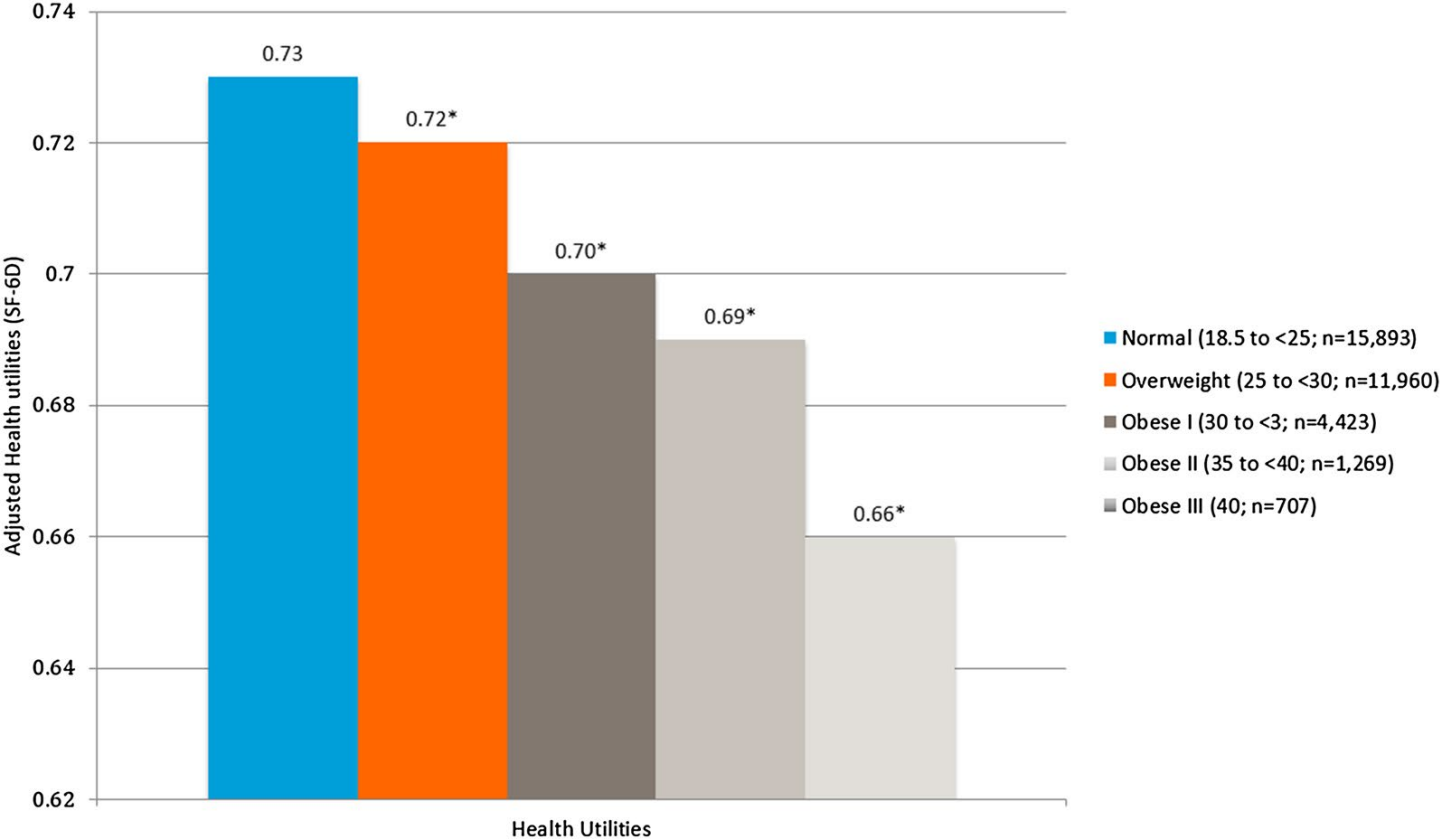
Adjusted mean health utilities (SF-36v2) by body mass index class among all respondents. Normal weight: 18.5–24.9 kg/m^2^; overweight: 25–29.9 kg/m^2^; obese class I: 30–34.9 kg/m^2^; obese class II: 35–39.9 kg/m^2^; obese class III: 40 kg/m^2^. *The mean is significantly different than normal-weight group. All models controlled for age, gender, race, marital status, education, income, exercise, smoking, alcohol use, and Charlson comorbidity index

Increasing BMI group was associated with significantly lower adjusted MCS, PCS, and SF-6D (Figs. 1, 2). For the normal BMI group the adjusted means for MCS was 47.21 and it was significantly higher than the overweight group (46.97; p < 0.05), obesity class I (45.91; p < 0.001), obesity class II (45.32; p < 0.001), and obesity class III (44.50; p < 0.001). For the PCS, the adjusted means for the normal BMI group was 52.05 and it was significantly higher than the overweight (51.38; p < 0.001), obesity class I (50.11; p < 0.001), obesity class II (48.27; p < 0.001), and obesity class III (46.02; p < 0.001). Additionally, the adjusted PCS surpassed the MID, the smallest change in an outcome that a patient would identify as important, for obesity classes II and III. For the SF-6D the adjusted means for the normal BMI group is 0.73. It was significantly higher than the overweight group (0.72; p < 0.001), obesity class I (0.70; p < 0.001), obesity class II (0.69; p < 0.001), and obesity class III (0.66).

#### Work productivity

**Fig. 3:**
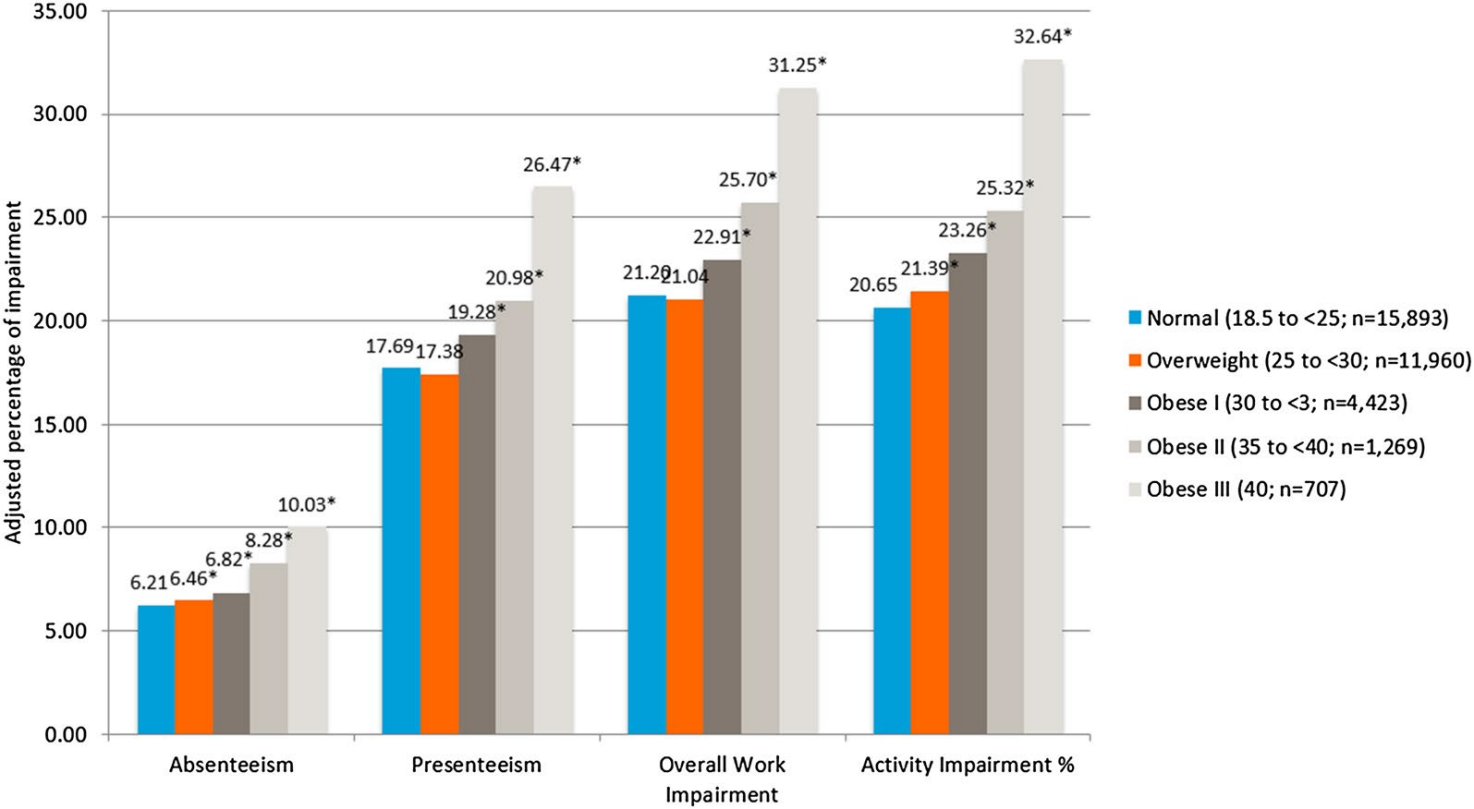
Adjusted for percentage of work productivity and activity impairment by body mass index class among all respondents. Normal weight: 18.5–24.9 kg/m^2^; overweight: 25–29.9 kg/m^2^; obese class I: 30–34.9 kg/m^2^; obese class II: 35–39.9 kg/m^2^; obese class III: 40 kg/m^2^. *The mean is significantly different than normal-weight group. All models controlled for age, gender, race, marital status, education, income, exercise, smoking, alcohol use, and Charlson comorbidity index

Virtually all the analyses involving the WPAI-GH found that increasing BMI group was positively associated with significantly greater impaired work productivity except for two instances; the scores of overweight participants were only slightly higher than those with a BMI in the normal range (Fig. 3). Specifically, for the normal BMI group the adjusted means for absenteeism was 6.21% and it was significantly lower than the overweight group (6.46; p = 0.02), obesity class I (6.82; p < 0.001), obesity class II (8.28; p < 0.001), and obesity class III (10.03; p < 0.001). For the normal BMI group the adjusted means for presenteeism was 17.69% and it was slightly higher, but not significantly different, than the overweight group (17.38%; p = 0.26). However, the normal weight group was significantly lower than obesity class I (19.28%; p = 0.001), obesity class II (20.98; p < 0.001), and obesity class III (26.47; p < 0.001). For the normal weight BMI group the adjusted mean for Overall Work Impairment was 21.20% and it was slightly higher, but not significantly different, than the overweight group (21.04; p = 0.62). However, the normal weight group was significantly lower than obesity class I (22.91%; p = 0.001), obesity class II (25.70; p < 0.001), and obesity class III (31.25; p < 0.001). The adjusted mean for the normal weight group for the activity impairment scale was 20.65 and it was significantly lower than the overweight group (21.39%; p < 0.001), obesity class I (23.26%; p < 0.001), obesity class II (25.32%; p < 0.001), and obesity class III (32.64%; p < 0.001).

#### Healthcare resource use

**Fig. 4:**
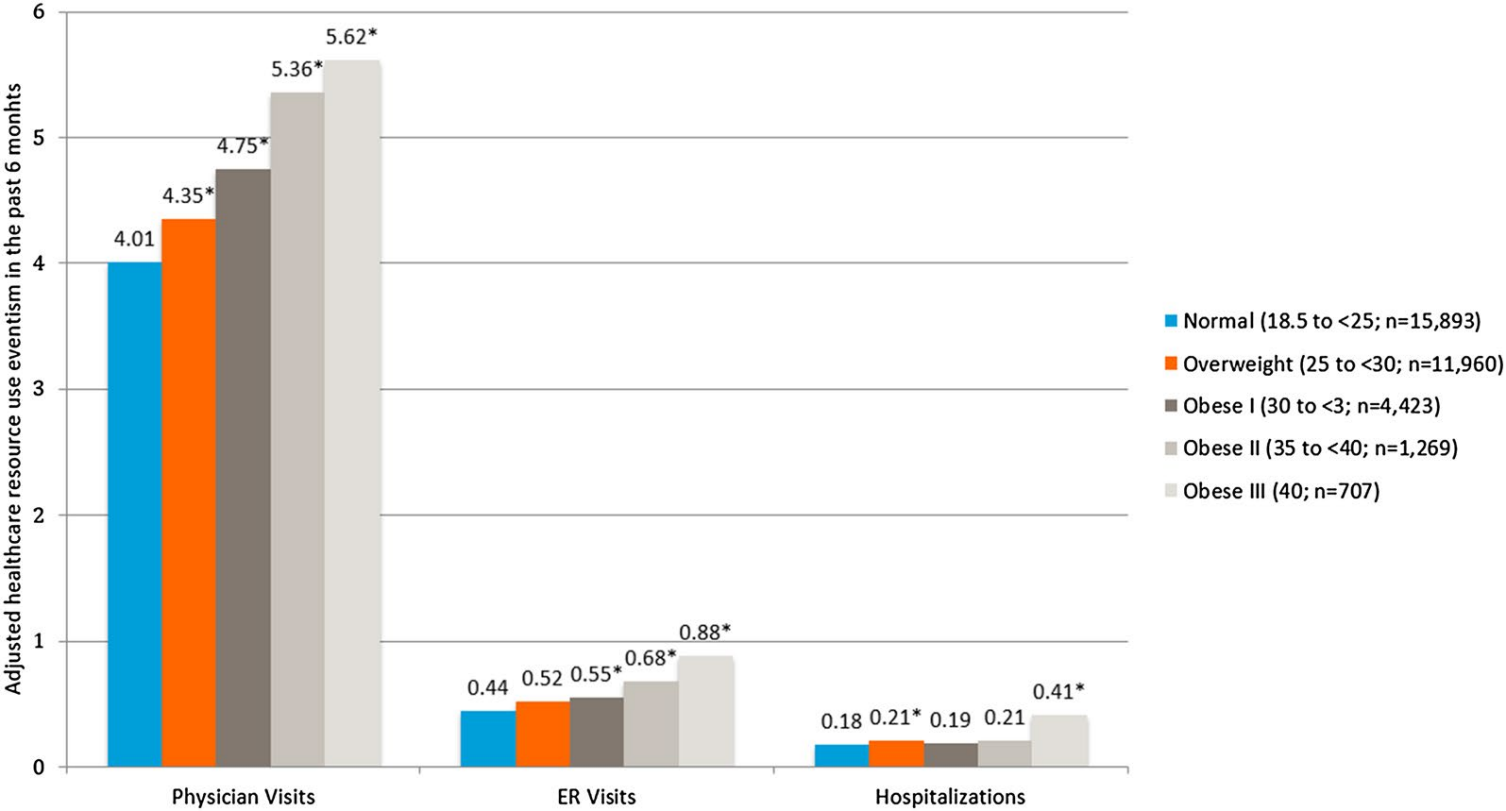
Adjusted mean for health care resource use in the past 6 months by body mass index class among all respondents. Normal weight: 18.5–24.9 kg/m^2^; overweight: 25–29.9 kg/m^2^; obese class I: 30–34.9 kg/m^2^; obese class II: 35–39.9 kg/m^2^; obese class III: 40 kg/m^2^. *The mean is significantly different than normal-weight group. All models controlled for age, gender, race, marital status, education, income, exercise, smoking, alcohol use, and Charlson comorbidity index

Analyses of the three HCRU variables found that increasing BMI group was generally associated with significantly greater provider visits, ER visits and hospitalizations. For the normal BMI group the adjusted means for physician visits was 4.01 and it was significantly lower than the overweight group (4.35; p < 0.001), obesity class I (4.75; p < 0.001), obesity class II (5.36; p < 0.001), and obesity class III (5.62; p < 0.001). For the normal BMI group the adjusted means for ER visits was 0.44 and it was significantly lower than the overweight group (0.52; p < 0.001), obesity class I (0.55; p < 0.001), obesity class II (0.68; p < 0.001), and obesity class III (0.88; p < 0.001). Analyses comparing hospitalizations found that the adjusted means for the normal BMI group was 0.18. This was significantly lower than the overweight group (0.21; p < 0.001), and obesity class III (0.41; p < 0.001), but it was not significantly different than obesity class I (0.19; p = 0.43), obesity class II (0.21; p = 0.06) (Fig. 4).

#### Costs

**Fig. 5:**
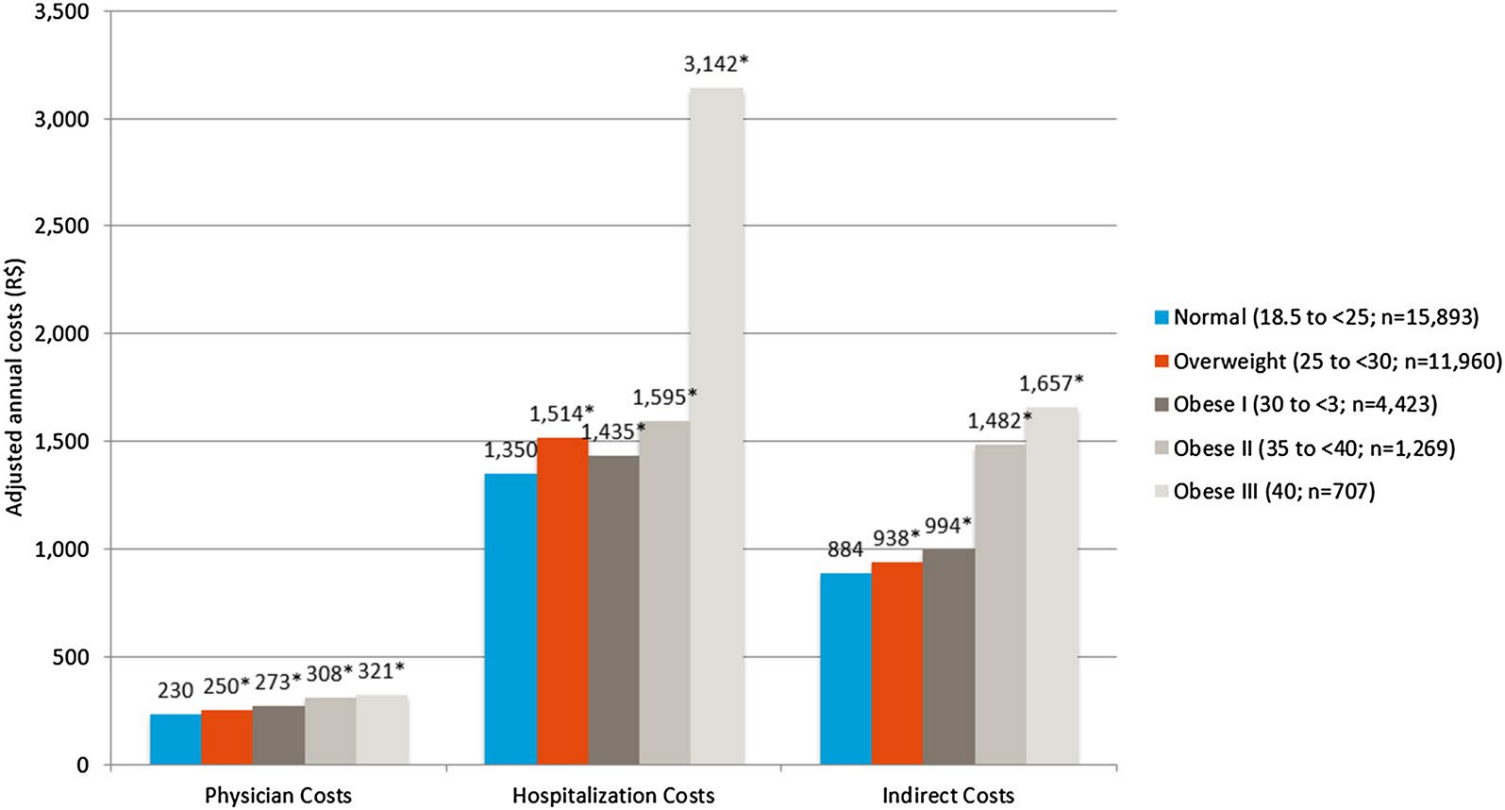
Adjusted annual cost differences across BMI classes among all adults. Normal weight: 18.5–24.9 kg/m^2^; overweight: 25–29.9 kg/m^2^; obese class I: 30–34.9 kg/m^2^; obese class II: 35–39.9 kg/m^2^; obese class III: 40 kg/m^2^. *The mean is significantly different than normal-weight group. All models controlled for age, gender, race, marital status, education, income, exercise, smoking, alcohol use, and Charlson comorbidity index

Finally, with regards to cost, increasing BMI group was associated with significantly greater physician, hospitalization, and indirect costs (Fig. 5). Multivariable analysis of the physician, hospitalizations, and indirect costs found that the normal weight group was significantly lower than all the other weight groups. For the normal BMI group the adjusted means for physician costs was R$230 and it was significantly lower than the overweight group (R$250; p < 0.001), obesity class I (R$273; p < 0.001) and obesity class II (R$308; p < 0.001). A comparison between the normal weight and obesity class III groups found that physician costs were 39.7% higher (R$321.22 vs R$229.94) and the differences were significant (p < 0.001). For the normal BMI group the adjusted means for hospitalization costs was R$1350 and it was significantly lower than the overweight (R$1514; p < 0.001), obesity class I (R$1435; p < 0.001), and obesity class II (R$1595; p < 0.001) groups. A comparison between normal weight and obese class III found hospitalization costs were over two times greater for the latter (R$1349.60 vs. R$3141.84) and significant (p < 0.001). For the indirect costs analyses the adjusted means for the normal weight group was R$884 and it was significantly lower than the overweight group (R$938; p < 0.001), obesity class I (R$994; p < 0.001), and obesity class II (R$1482; p < 0.001). A comparison between indirect costs found that that normal weight group was almost half of those for the Obesity Class III group (R$884.15 vs. R$1656.80) and they were significantly different (p < 0.001).

## Discussion

Over half (53.6%) the adults surveyed possessed a higher than normal BMI, which is consistent with previous studies [31]. Our results demonstrate that increasing BMI group is associated with progressively worsening health status, which is consistent with prior findings that demonstrate that obesity most strongly affects physical domains of functioning [17, 20]. The analyses also noted an association between increasing BMI group and both greater healthcare resource use and costs. Specifically, the cost of the work productivity loss for those in the obesity class III group, compared to normal weight respondents, is over 50% higher. These findings were also consistent with prior research [19]. Further, a significant association was also observed between increasing BMI group and greater healthcare resource utilization and direct costs, as suggested in studies conducted in other countries [19, 20].

This study, one in a long line of many conducted throughout the world, continues to demonstrate the humanistic and societal cost associated with obesity [22, 25, 41–45]. Obesity is associated with increased direct costs [29, 30, 46], poor mental function including binge eating, anxiety, and depression [47], and indirect costs [42]. This study, even though it relies exclusively on self-report data, supports these findings [31]. Many causes have been attributed to escalating prevalence of obesity including economic growth, urbanization, sedentary lifestyle, and increasing use of processed foods and high calorie diets [41]. Not surprisingly, it is expected that the obesity rates in Brazil will continue to climb through 2050 [31] and thus the incidence of coronary heart disease, stroke, hypertension, cancers, osteoarthritis, and diabetes are projected to at least double by 2050 too. Surgical [48], behavioral [49], exercise [50], pharmacotherapy [51], and workplace [52] interventions have been found to effectively reduce weight, which might help to impact these trends.

### Limitations

Although the findings presented in this study generally consistent with the previous obesity-related research, there are some limitations that should be noted. First, all data were self-reported and no verification from patients’ medical charts or other objective confirmation of BMI class, health history information or healthcare resource use was collected. It is also possible that reporting errors based on recall may have thus occurred. Furthermore, technology limitations may have biased the sample to favor younger, more educated, healthier adults, hence under-representing the those very sick, bedridden, or hospitalized who could not complete a 30-min online survey or those not able to obtain access to a computer or the internet due to cost, availability or other reasons. Additionally, this is a cross-sectional study and thus no causal claim can be made. Further, estimated costs may be different than true costs obtained through other means. Finally, disability-related costs and other non-wage related variables were not accounted for in the indirect cost calculation. Finally, although the NHWS is demographically representative of the general Brazilian adult population with respect to age, sex, and socioeconomic status, it is unclear to what extent this sample generalizes to the specific population of obese adults or whether the sample accurately represents the characteristics of workers within each major occupational category examined.

### Conclusions

The current study found that obesity rates in Brazil are considerable and, from a patient and societal perspective, increasingly burdensome. The results are consistent with previous research and highlight the need for Brazilian policy makers, healthcare providers, and all other stakeholders to prioritize strategies for weight management.

## Abbreviations

BMI: body mass index
CBHPM: Classificação Brasileira Hierarquizada de Procedimentos Médicos
CCI: Charlson comorbidity index
ER: emergency room
GLM: generalized linear model
HRQoL: health-related quality of life
IRB: Institutional Review Board
MCS: mental component summary
MID: minimally important difference
NHWS: National Health and Wellness Survey
PCS: physical component summary
SAS: Secretaria de Atenção à Saúde
SD: standard deviation
SF-6D: Short Form 6-Dimensions
SF-12: Medical Outcomes Study 12-Item Health Survey
SF-36: Medical Outcomes Study 36-Item Health Survey
SIGTAP: Sistema de Gerenciamento da Tabela de Procedimentos, Medicamentos e OPM do
SIH/SUS; AIH: Sistema de Informações Hospitalares no SUS
UNIDAS: União Nacional das Instituições de Autogestão em Saúde
US: United States
WPAI-GH: Work Productivity and Activity Impairment-General Health Questionnaire
